# Body mass index related electrocardiographic findings in healthy young individuals with a normal body mass index

**DOI:** 10.1007/s12471-019-1282-x

**Published:** 2019-05-20

**Authors:** G. J. Hassing, H. E. C. van der Wall, G. J. P. van Westen, M. J. B. Kemme, A. Adiyaman, A. Elvan, J. Burggraaf, P. Gal

**Affiliations:** 1 Centre for Human Drug Research, Leiden, The Netherlands; 2 Leiden Academic Centre for Drug Research, Leiden, The Netherlands; 3 Department of Cardiology, VU Medical Center, Amsterdam, The Netherlands; 4 Department of Cardiology, Isala Hospital, Zwolle, The Netherlands; 5 Leiden University Medical Center, Leiden, The Netherlands

**Keywords:** BMI, ECG, Healthy, Obesity

## Abstract

**Introduction:**

An increased body mass index (BMI) (>25 kg/m^2^) is associated with a wide range of electrocardiographic changes. However, the association between electrocardiographic changes and BMI in healthy young individuals with a normal BMI (18.5–25 kg/m^2^) is unknown. The aim of this study was to evaluate the association between BMI and electrocardiographic parameters.

**Methods:**

Data from 1,290 volunteers aged 18 to 30 years collected at our centre were analysed. Only subjects considered healthy by a physician after review of collected data with a normal BMI and in sinus rhythm were included in the analysis. Subjects with a normal BMI (18.5–25 kg/m^2^) were divided into BMI quartiles analysis and a backward multivariate regression analysis with a normal BMI as a continuous variable was performed.

**Results:**

Mean age was 22.7 ± 3.0 years, mean BMI was 22.0, and 73.4% were male. There were significant differences between the BMI quartiles in terms of maximum *P*-wave duration, *P*-wave balance, total *P*-wave area in lead V1, PR-interval duration, and heart axis. In the multivariate model maximum *P*-wave duration (standardised coefficient (SC) = +0.112, *P* < 0.001), *P*-wave balance in lead V1 (SC = +0.072, *P* < 0.001), heart axis (SC = −0.164, *P* < 0.001), and Sokolow-Lyon voltage (SC = −0.097, *P* < 0.001) were independently associated with BMI.

**Conclusion:**

Increased BMI was related with discrete electrocardiographic alterations including an increased *P*-wave duration, increased *P*-wave balance, a leftward shift of the heart axis, and decreased Sokolow-Lyon voltage on a standard twelve lead electrocardiogram in healthy young individuals with a normal BMI.

## What’s new

- Obesity-associated electrocardiographic changes are also found in healthy young individuals with a normal body mass index (BMI) (18.5–25 kg/m^2^).
- There were significant differences between the BMI quartiles in terms of maximum *P*-wave duration, *P*-wave balance, total *P*-wave area in lead V1, PR-interval duration, and heart axis.
- Within the normal range, an increased BMI was independently associated with an increased *P*-wave duration, increased *P*-wave balance, a leftward shift of the heart axis, and decreased Sokolow-Lyon voltage.

## Introduction

Obesity causes several haemodynamic changes such as increased blood and stroke volume, and an increase in pulmonary and left atrial pressure [1, 2]. These changes cause structurally altered cardiac tissue such as left atrial enlargement and remodelling, and ventricular hypertrophy [1, 2]. These may ultimately result in obesity-induced left ventricular diastolic and systolic dysfunction and right and left ventricular heart failure [1, 2].

Some obesity-induced adverse effects on cardiac function can be identified on a 12-lead electrocardiogram (ECG). This includes an increased *P*-wave duration and dispersion [3–6], prolongation of the PR interval [3–7], low QRS voltage in the limb leads [7–9], leftward shift of the heart axis [7–11], various markers of left ventricular hypertrophy [12–14] and prolongation of the corrected QT interval and prolonged QT-interval duration [8]. Many of these electrocardiographic abnormalities have been reported to be reversible with substantial weight loss thereby reinforcing the association between BMI and electrocardiographic changes [2, 8].

These electrocardiographic changes are well-documented in obese individuals. However, to which extent these electrocardiographic changes are associated with BMI in healthy young individuals with a normal BMI (18.5–25 kg/m^2^) is largely unknown. In addition, subtle physiological changes in these individuals are of particular interest in early phase pharmaceutical research because they help differentiate between normal physiological changes or potentially harmful or unknown pharmacodynamic effects. The aim of the present analysis was to evaluate the association between BMI and selected electrocardiographic parameters related to cardiac alterations in obesity in a healthy young population with a normal BMI.

## Methods

Data from 1,290 male and female volunteers with a normal BMI (18.5–25.0 kg/m^2^) aged 18–30 years used in the present analysis were collected at the Centre for Human Drug Research in Leiden, the Netherlands, a clinical research organisation specialised in early-phase drug development studies. Data from studies that were performed in healthy volunteers between 2010 and 2016 were included in the present analysis. For all studies, healthy volunteers underwent a mandatory medical screening to verify eligibility for the study. The present analysis was performed in accordance to local regulations. All activities were performed in accordance to applicable standard operating procedures.

## Medical screening

The medical screening consisted of a single visit to the clinical unit where a detailed medical history, a physical examination, vital signs including blood pressure, temperature, weight and height measurement, BMI calculation, and a twelve-lead electrocardiogram were recorded. Additionally, haematology and chemistry blood panel, urine dipstick, and a urine drug test were recorded.

## Weight and height measurement

For the weight and height measurement, the subject was asked to undress except for underwear and asked to stand on the platform with the back against the measuring rod, heels against the heel board and back and neck straight. A BMI was calculated automatically by the digital scale using the formula:

$$\mathrm{BMI}=\frac{\text{Weight}\,\left(\text{kilograms}\right)}{\text{Height}\,\left(\text{metres}\right)^{2}}$$

Body weight and height measurements were recorded with a calibrated digital measuring rod (SECA 285; RevaMed BV, Kampen, the Netherlands) and immediately entered into a validated database system (Promasys, OmniComm, Fort Lauderdale, FL, USA).

## ECG measurements

The twelve-lead ECGs were recorded with the volunteer in a resting supine position and after a five-minute resting period. The twelve-lead ECGs were recorded using an electrocardiograph (Marquette 800/5500/2000 or Dash 3000; General Electric Healthcare, Milwaukee, USA) and twelve disposable electrodes placed in the standard anatomical position. The ECG data were then uploaded into the ECG warehouse (Muse Cardiology Data Management System v7, General Electric Healthcare, Chicago, IL, USA). The Marquette Cubic Spline and Finite Residual Filtering filters were used for artefact and noise management. The ECG warehouse automatically assesses interval and amplitude data from the digital ECGs with the Marquette 12SL algorithm, which provides a variety of ECG measurements which have been used in previous studies [15, 16]. In addition, a physician reviewed all ECGs for quality, legibility and abnormalities. Independent evaluation showed that the Marquette 12SL algorithm passed all of the amplitude measurement requirements (maximum of 10 ms deviation) as defined in International Electrotechnical Commission, as described in the GE Physician’s Guide (version 2036070-006). Description, methods of determination and calculation, and units of the electrocardiographic parameters are described in Tab. 1.

**Table 1:** Methods of determination or calculation of the electrocardiographic (ECG) variables used in the analysis of the 1,554 healthy volunteers aged 18 to 30 years with a body mass index (BMI) between 18.5 and 30.0 kg/m^2^

Variable	Description
Maximum *P*-wave duration (ms)	Longest *P*-wave duration sampled from all leads
*P*-wave balance in lead V1 (µV)	Difference between the upward and downward deflection of the *P*‑wave
*P*-wave dispersion (ms)	Difference between the longest minus the shortest *P*-wave duration from all leads
Total *P*-wave area in lead V1 (mm*ms)	Sum of the total area under and above the isoelectric line from onset to termination of the *P*‑wave [27]
PR interval (ms)	Beginning of the *P*‑wave until the beginning of the QRS complex
QRS duration (ms)	First deflection from the isoelectric line following the *P*‑wave until the J‑point
Heart axis (degrees)	Net vector of the R wave axis based on the extremity leads
Sokolow-Lyon voltage (mm)	Sum amplitude of the S wave in lead V1 and the amplitude of the R wave in lead V5 or V6 (whichever is larger) [28]
Cornell product (ms*mm)	Product of the QRS duration and the Cornell voltage [29]. Cornell voltage is the sum of the amplitude of the R wave in lead aVL and the amplitude of the S wave in lead V3 [29]
Maximum T‑wave duration (ms)	Longest T‑wave duration sampled from all leads
Minimum T‑wave duration (ms)	Shortest T‑wave duration sampled from all leads
T-wave dispersion (ms)	Difference between the longest and shortest T‑wave duration selected from all leads
QTcF duration (ms)	QTcF duration is calculated using the Fridericia formula, which divides the QT interval by the cube root of RR interval [30]. QT interval is the interval between the start of the Q wave and the end of the T wave. RR interval is the interval between the onset of one QRS complex to the onset of the next QRS complex, measured in seconds, derived from the heart rate (HR) as 60/HR

*mV* millivolt, *µV* microvolt, *ms* milliseconds, *mm* millimetres, *QTcF* corrected QT interval with the Fridericia formula

## Validation cohort

Additionally, data from 255 male and female volunteers with an overweight BMI (25.1–30.0 kg/m^2^) aged 18–30 years similarly collected as the data from volunteers with a normal BMI (18.5–25.0 kg/m^2^) were added to the analysis as a validation cohort. These data was used to test the persistence of any significant variances of ECG findings in the healthy subjects with a normal BMI (18.5–25.0 kg/m^2^) in that of healthy subjects with an overweight BMI (25.1–30.0 kg/m^2^). This data was not included in the univariate and multivariate analysis.

## Statistical analysis

Data are reported as mean ± standard deviation (SD), median with interquartile range or percentage where appropriate. Categorical variables were compared using chi-squared test. Variances were compared using the Analysis of Variance (ANOVA) test with a post-hoc Tukey analysis. A linear univariate and a backward linear multivariate regression model analysis were performed solely with the data of the subjects with a normal BMI (18.5–25.0 kg/m^2^). Probabilities of less than 0.10 in the linear univariate regression model were added to the backward linear multivariate regression model. Results are reported as unstandardised coefficient (USC) and standardised coefficient (SC) with the corresponding *P* value. Statistical analyses were performed using IBM SPSS version 25 (IBM corporation, Armonk, NY, USA).

## Results

In total, 1,290 subjects were included in the present analysis. Mean age was 22.6 ± 3.0 years, 73.9% were male. Subjects with a normal BMI (*n* = 1,290) were divided based on BMI quartiles (18.5–20.7; 20.7–22.0; 22.0–23.4; 23.4–25.0 kg/m^2^). Overweight subjects (*n* = 255) were allocated in to the overweight group. Subject characteristics are shown in Tab. 2. Subjects in the lowest BMI quartile were significantly younger and had a significantly lower systolic blood pressure compared with subjects in the third (*P* = 0.003 and *P* < 0.001 respectively) and fourth BMI quartile (*P* < 0.001 and *P* < 0.001 respectively). In addition, overweight subjects were significantly younger than the first quartile (*P* = 0.003), and had a significantly higher systolic blood pressure compared with subjects in the first (*P* < 0.001), second (*P* < 0.001), and third BMI quartile (*P* = 0.006). Other baseline characteristics were not significantly different among BMI groups.

**Table 2:** Relation between patient characteristics and electrocardiographic parameters to BMI of included healthy volunteers

	Body mass index (kg/m^2^)				
	18.5–20.7 (*n* = 323)	20.8–22.0 (*n* = 334)	22.1–23.4 (*n* = 324)	23.5–25.0 (*n* = 309)	25.1–30.0 (*n* = 255)
Corresponding group	*α*	*ß*	*γ*	*δ*	*ε*
Age (years)	22.2 ± 2.93 ^γ δ ε^	22.6 ± 3.02	23.0 ± 2.93 ^α^	23.1 ± 3.05 ^α^	23.1 ± 2.89 ^α^
Gender (% male)	70.0	72.8	76.5	75.4	77.5
Temperature (°C)	36.7 ± 0.35	36.7 ± 0.40	36.7 ± 0.40	36.7 ± 0.39	36.8 ± 0.39
Systolic blood pressure (mm Hg)	117 ± 10.2 ^γ δ ε^	119 ± 10.5 ^ε^	120 ± 9.63 ^α ε^	121 ± 10.0 ^α^	123 ± 10.0 ^α ß γ^
Diastolic blood pressure (mm Hg)	67.9 ± 7.77	68.3 ± 7.74	68.6 ± 7.68	68.4 ± 7.50	69.1 ± 8.34
Ventricular rate (beats/min)	64.4 ± 9.99	62.7 ± 9.69	62.5 ± 10.6	62.6 ± 9.83	64.7 ± 10.6
Serum Sodium (mmol/l)	141 ± 1.82	141 ± 1.90	141 ± 1.92	141 ± 1.85	141 ± 1.91
Serum Potassium (mmol/l)	4.31 ± 0.33	4.33 ± 0.31	4.33 ± 0.29	4.36 ± 0.30	4.33 ± 0.30
Serum Calcium (mmol/l)	2.42 ± 0.09	2.42 ± 0.09	2.41 ± 0.09	2.42 ± 0.09	2.42 ± 0.10
Maximum *P*-wave duration (ms)	99.09 ± 10.4 ^δ ε^	100.5 ± 11.5 ^δ ε^	101.2 ± 10.7 ^ε^	102.9 ± 10.6 ^α ß^	103.8 ± 11.1 ^α ß γ^
*P*-wave balance in lead V1 (µV)	0.31 ± 0.45 ^δ ε^	0.32 ± 0.42 ^δ ε^	0.33 ± 0.43	0.41 ± 0.41 ^α ß^	0.42 ± 0.35 ^α ß^
*P*-wave dispersion (ms)	51.5 ± 20.8	53.6 ± 19.9	52.0 ± 21.0	51.7 ± 21.3	50.9 ± 20.9
Total *P*-wave area in lead V1 (mm*ms)	47.53 ± 82.6 ^δ ε^	47.15 ± 76.3 ^δ ε^	50.50 ± 79.5 ^δ ε^	67.91 ± 80.0 ^α ß γ^	70.96 ± 74.0 ^α ß γ^
PR interval (ms)	146.9 ± 18.1 ^γ δ ε^	149.9 ± 21.9	152.2 ± 21.0 ^α^	151.5 ± 20.7 ^α^	153.1 ± 19.0 ^α^
QRS duration (ms)	96.0 ± 10	96.4 ± 10	97.7 ± 10	97.0 ± 10	96.9 ± 10
Heart axis (degrees)	68.65 ± 25.8 ^γ δ ε^	63.90 ± 26.0 ^δ ε^	59.77 ± 27.0 ^α ε^	54.57 ± 28.3 ^α ß^	51.91 ± 28.2 ^α ß γ^
Sokolow-Lyon voltage (mm)	28.02 ± 8.28	27.30 ± 7.78	27.83 ± 8.27	26.80 ± 7.69	26.77 ± 7.66
Cornell product (mm*µV)	11.45 ± 6.29	12.29 ± 6.52	12.47 ± 6.21	11.74 ± 5.78	12.07 ± 6.09
Maximum T‑wave duration (ms)	183 ± 20	184 ± 20	182 ± 20	183 ± 20	181 ± 21
Minimum T‑wave duration (ms)	107 ± 54	115 ± 52	110 ± 49	114 ± 51	109 ± 50
T-wave dispersion (µV)	75.9 ± 54.7	69.4 ± 52.9	72.5 ± 54.7	68.3 ± 51.8	72.4 ± 50.5
QTcF duration (ms)	404.8 ± 17	406.7 ± 18	405.4 ± 18	405.1 ± 19	404.0 ± 19

Categorical variables were compared using chi-squared test, variances were compared using the Analysis of Variance test with a post hoc Tukey analysis. Results are reported as mean ± SD or as percentage. The symbols α, ß, γ, δ, and ε represent a significant difference (*P* < 0.05) compared with that group. If no symbols are present, no significance was found between the groups

*mV* millivolt, *µV* microvolt, *ms* milliseconds, *mm* millimetres

## BMI and electrocardiographic parameters

**Fig. 1:**
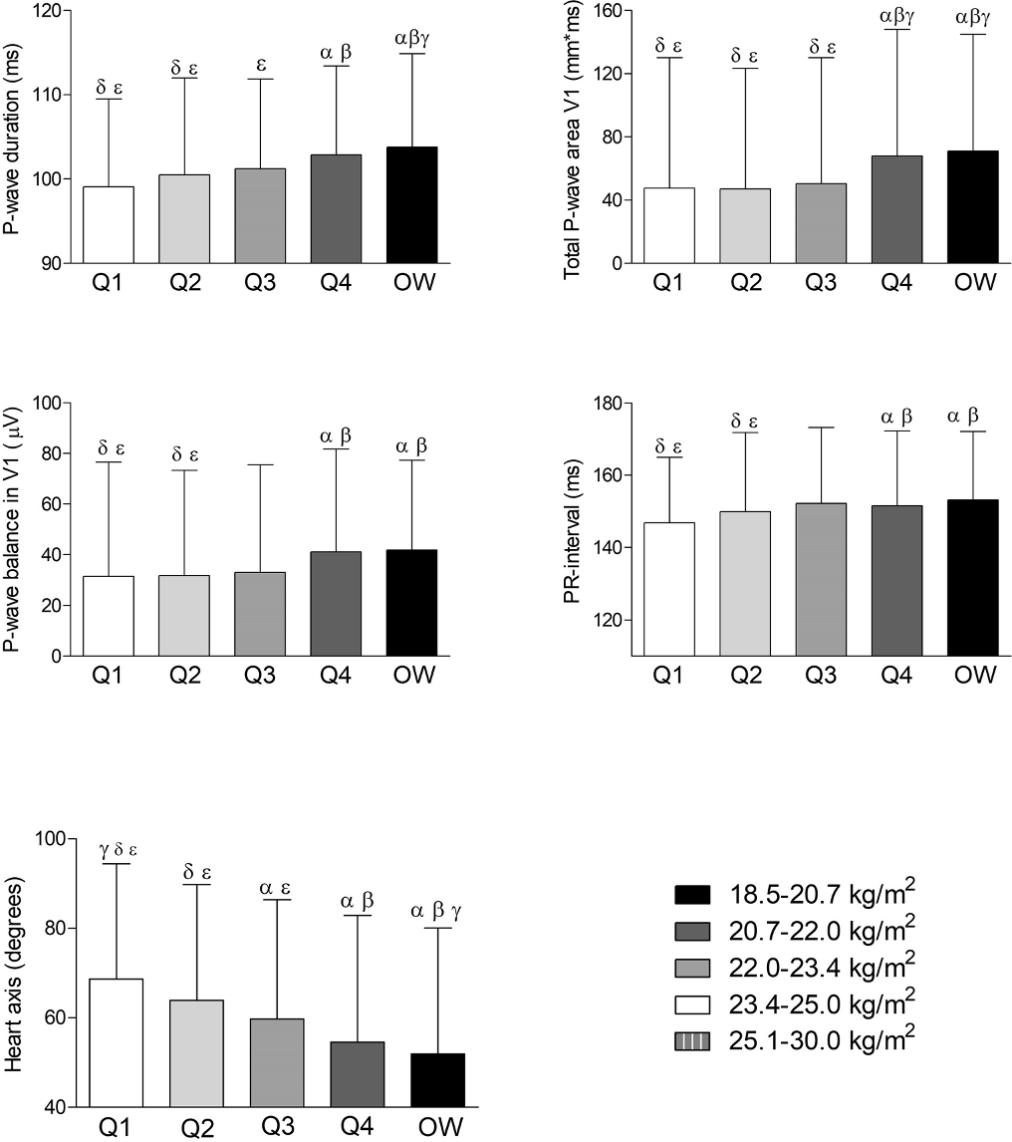
Associations of body mass index (BMI) with electrocardiographic parameters. Results were based on Analysis of Variance test between BMI distribution, and expressed as difference in the electrocardiographic parameter (with 95% confidence interval) per quartile of BMI using a post hoc Tukey analysis. The symbols α, ß, γ, δ, and ε represent a significant difference (*P* < 0.05) compared with that group. If no symbols are present, no significance was found between the groups. *OW* overweight BMI (25.1–30.0 kg/m^2^) group, *µV* microvolt, *ms* milliseconds, mm*ms millimetre times milliseconds

Table 2 displays the association between the BMI quartiles and the evaluated electrocardiographic parameters. Maximum *P*-wave duration, *P*-wave balance, total *P*-wave area in lead V1, PR interval, and heart axis were significantly different between the normal BMI quartiles and the overweight BMI group, as displayed in Fig. 1.

## Linear regression analysis

In the univariate analysis of the data of the subjects with a normal BMI (18.5–25.0 kg/m^2^), BMI was significantly associated (*P* < 0.05) with age (SC = +0.139, *P* < 0.001), systolic blood pressure (SC + 0.146, *P* < 0.001), ventricular rate (SC = −0.076, *P* = 0.006), serum creatinine (SC = +0.152, *P* < 0.001), serum potassium (SC = +0.054, *P* = 0.054), maximum *P*-wave duration (SC = +0.130, *P* < 0.001), *P*-wave balance in lead V1 (SC = +0.077, *P* = 0.006), total *P*-wave area in lead V1 (SC = +0.084, *P* = 0.003), PR interval (SC = +0.090, *P* = 0.001), and heart axis (SC = −0.191, *P* < 0.001). In the backward multivariate model, age (SC = +0.108, *P* < 0.001), systolic blood pressure (SC = +0.173, *P* < 0.001), ventricular rate (SC = −0.081, *P* = 0.005), maximum *P*-wave duration (SC = +0.112, *P* < 0.001), *P*-wave balance in lead V1 (SC = +0.072, *P* < 0.001), heart axis (SC = −0.164, *P* < 0.001), and Sokolow-Lyon voltage (SC = −0.097, *P* < 0.001) were independently associated with BMI, as can also be observed from Tab. 3. The R square of the multivariate model was 0.100.

**Table 3:** Univariate and backward linear multivariate regression model analysis

	Univariate analysis			Multivariate analysis			
Variable	USC	SC	R square	*P* value	USC	SC	*P* value
Age (per year)	0.079	0.139	0.019	<0.001	0.061	0.108	<0.001
Female gender	−0.211	−0.054	0.003	0.056	Excluded		
Temperature (°C)	0.038	0.009	0.000	0.764			
Systolic blood pressure (mm Hg)	0.024	0.146	0.021	<0.001	0.029	0.173	<0.001
Diastolic blood pressure (mm Hg)	0.005	0.022	0.000	0.479			
Ventricular rate (beats/min)	−0.013	−0.076	0.006	0.006	−0.014	−0.081	0.005
Serum Sodium (mmol/l)	−0.022	−0.024	0.001	0.399			
Serum Potassium (mmol/l)	0.299	0.054	0.003	0.054	0.253	0.046	0.098
Serum Calcium (mmol/l)	−0.735	−0.038	0.001	0.178			
Maximum *P*-wave duration (ms)	0.020	0.130	0.017	<0.001	0.018	0.112	<0.001
*P*-wave balance in lead V1 (µV)	0.003	0.077	0.006	0.006	0.003	0.072	0.010
*P*-wave dispersion (ms)	0.001	0.009	0.000	0.740			
Total *P*-wave area in lead V1 (mm*ms)	0.002	0.084	0.007	0.003	Excluded		
PR interval (ms)	0.007	0.090	0.008	0.001	Excluded		
QRS duration (ms)	0.009	0.053	0.003	0.059	Excluded		
Heart axis (degrees)	−0.012	−0.191	0.036	<0.001	−0.010	−0.164	<0.001
Sokolow-Lyon voltage (mm)	0.000	−0.048	0.002	0.084	0.000	−0.097	<0.001
Cornell product (mm)	5.67 *10^−7^	0.021	0.000	0.458			
Maximum T‑wave duration (ms)	−0.002	−0.019	0.000	0.503			
Minimum T‑wave duration (ms)	0.001	0.038	0.001	0.171			
T-wave dispersion (ms)	−0.001	−0.045	0.002	0.107			
QTcF (ms)	5.38 *10^−5^	0.001	0.000	0.982			

Probabilities of less than 0.10 in the linear univariate regression model were added to the backward linear multivariate regression model. Results are reported as unstandardised coefficient (USC) and standardised coefficient (SC) with the corresponding *P* value and the R‑square value in the linear univariate regression model. The R square of the backward linear multivariate regression model was 0.100

*mV* millivolt, *µV* microvolt, *ms* milliseconds, *mm* millimetres, *QTcF* corrected QT interval with the Fridericia formula

## Discussion

This analysis found an association between electrocardiographic parameters and BMI in healthy young (≤ 30 years) adults with a normal BMI (18.5–25.0 kg/m^2^). A higher BMI was independently associated with an increased *P*-wave duration, an increased *P*‑wave, a leftward shift of the heart axis, and a decreased Sokolow-Lyon voltage.

Left atrial enlargement (LAE) is associated with an increased prevalence of atrial fibrillation, cardiovascular events and death [13]. Obesity is found to be the most important risk factor for LAE development in the general population [17], and is dependent on the extent of obesity [2–6]. Furthermore, LAE is also independently related to age, hypertension, BMI, waist circumference, and metabolic syndrome [18], Additionally, obesity is the strongest predictor of LAE in hypertensive patients, and is under the influence of race and gender [19]. These structural changes can be observed on the twelve-lead surface ECG through increased *P*-wave duration, *P*-wave area, and *P*-wave dispersion [2–6]. Obesity-associated electrocardiographic changes such as an increased *P*-wave duration (5–22 ms) and *P*-wave dispersion (14–25 ms) [3–6], increased PR interval (5–13 ms) [3–7] and a leftward shift of the heart axis (11–37 degrees) compared with adults with a normal BMI were reported [7–11]. In the present analysis, we found a relation between BMI and these indices of atrial size. Although no left atrial measurement was performed these results suggest that atrial size may also be related to BMI in healthy individuals with a normal BMI (18.5–25.0 kg/m^2^).

Presumably, increased epicardial and pericardial fat, which are increased in obesity, further induce these changes [2, 20–25]. Cardiac fat depositions were found to have metabolic and inflammatory functions which can contribute to the fibrotic remodelling of the atrial tissue [1, 2, 20–25]. These fat depositions are significantly increased in obesity and are believed to induce the abovementioned electrocardiographic changes [1, 2, 20–25]. Hypothetically, the volume of epicardial and pericardial fat is also dependent on BMI in young, non-obese individuals. This may be an additional explanation for the association that was observed in the present analysis between BMI and the above-mentioned electrocardiographic changes.

Leftward shifts of the *P*-wave, QRS and T‑wave axes (11–37 degrees) are reported in obese patients compared with healthy controls [7–11]. The cause of these shifts is uncertain, but may be related to a leftward and more horizontal orientation of the heart attributed to the diaphragmatic pressure from central obesity, independent from left ventricular hypertrophy [7–11]. This explains the association between lower BMI and rightward *P*-wave and QRS axes and independently from left ventricular mass [11]. This is in line with our findings and presumably, the leftward change in heart axis that was observed in the present analysis is caused by an increase in diaphragmatic pressure which is dependent on BMI.

Previous reports already advocated caution when using ECG markers for left ventricular hypertrophy (LVH) in obese patients [14]. The commonly used Sokolow-Lyon voltage criteria underestimate the prevalence of anatomic LVH in the presence of obesity, whereas Cornell product criteria for ECG LVH appear to provide a more accurate measure of LVH in obese and overweight patients [12]. Obesity results in three distinct processes that affect the surface ECG—lateral displacement of the anatomical left ventricular (LV) axis, increased chest wall fat and increased pericardial fat mass—all of which decrease voltage amplitude on the ECG [26]. We found decreased Sokolow-Lyon voltages in the multivariate analysis, but no associations with the Cornell product calculations. These findings illustrate the challenge of their use as marker for LVH even in healthy individuals with a normal BMI (18.5–25.0 kg/m^2^).

## Limitations

The limitations of this study are the retrospective, cross-sectional design, and the usage of automatically calculated ECG data. The reported associations were found to be significant in the multivariate model, however the R square of the multivariate model was only modest, suggesting that other co-existing factors play a role in atrial and ventricular structural and functional remodelling. Larger prospective cohort studies are needed to explore the prognostic value of these ECG findings. Additional information such as waist circumference, cardiac dimensions, and more detailed information about the body composition such as fat and muscle percentages may further differentiate between groups and provide new insights about the cardiac changes.

## Conclusion

In conclusion, we found that BMI-related discrete electrocardiographic changes can be observed in healthy young individuals with a normal BMI (18.5–25.0 kg/m^2^). These were related to an altered atrial conduction, leftward shift of the heart axis, and decreased Sokolow-Lyon voltage.
